# Primary Drug-Resistant Tuberculosis in Hanoi, Viet Nam: Present Status and Risk Factors

**DOI:** 10.1371/journal.pone.0071867

**Published:** 2013-08-13

**Authors:** Nguyen Thi Le Hang, Shinji Maeda, Luu Thi Lien, Pham Huu Thuong, Nguyen Van Hung, Tran Bich Thuy, Akiko Nanri, Tetsuya Mizoue, Nguyen Phuong Hoang, Vu Cao Cuong, Khieu Thi Thuy Ngoc, Shinsaku Sakurada, Hiroyoshi Endo, Naoto Keicho

**Affiliations:** 1 NCGM-BMH Medical Collaboration Center, Hanoi, Viet Nam; 2 Department of Mycobacterium Reference and Research, Research Institute of Tuberculosis JATA, Tokyo, Japan; 3 Hanoi Department of Health, Hanoi, Viet Nam; 4 Hanoi Lung Hospital, Hanoi, Viet Nam; 5 Department of Microbiology, National Lung Hospital, Hanoi, Viet Nam; 6 Department of Epidemiology and Prevention, Clinical Research Center, National Center for Global Health and Medicine, Tokyo, Japan; 7 Department of Microbiology, Hanoi Lung Hospital, Hanoi, Viet Nam; 8 Bureau of International Medical Cooperation, National Center for Global Health and Medicine, Tokyo, Japan; 9 Department of International Affairs and Tropical Medicine, Tokyo Women’s Medical University, Tokyo, Japan; 10 Department of Pathophysiology and Host Defense, Research Institute of Tuberculosis JATA, Tokyo, Japan; 11 National Center for Global Health and Medicine, Tokyo, Japan; St. Petersburg Pasteur Institute, Russian Federation

## Abstract

**Introduction:**

Resistance of *Mycobacterium tuberculosis* (MTB) to anti-tuberculosis (TB) drugs presents a serious challenge to TB control worldwide. We investigated the status of drug resistance, including multidrug-resistant (MDR) TB, and possible risk factors among newly diagnosed TB patients in Hanoi, the capital of Viet Nam.

**Methods:**

Clinical and epidemiological information was collected from 506 newly diagnosed patients with sputum smear- and culture-positive TB, and 489 (96.6%) MTB isolates were subjected to conventional drug susceptibility testing, spoligotyping, and 15-locus variable numbers of tandem repeats typing. Adjusted odds ratios (aORs) were calculated to analyze the risk factors for primary drug resistance.

**Results:**

Of 489 isolates, 298 (60.9%) were sensitive to all drugs tested. Resistance to isoniazid, rifampicin, streptomycin, ethambutol, and MDR accounted for 28.2%, 4.9%, 28.2%, 2.9%, and 4.5%, respectively. Of 24 isolates with rifampicin resistance, 22 (91.7%) were MDR and also resistant to streptomycin, except one case. Factors associated with isoniazid resistance included living in old urban areas, presence of the Beijing genotype, and clustered strains [aOR = 2.23, 95% confidence interval (CI) 1.15–4.35; 1.91, 1.18–3.10; and 1.69, 1.06–2.69, respectively). The Beijing genotype was also associated with streptomycin resistance (aOR = 2.10, 95% CI 1.29–3.40). Human immunodeficiency virus (HIV) coinfection was associated with rifampicin resistance and MDR (aOR = 5.42, 95% CI 2.07–14.14; 6.23, 2.34–16.58, respectively).

**Conclusion:**

Isoniazid and streptomycin resistance was observed in more than a quarter of TB patients without treatment history in Hanoi. Transmission of isoniazid-resistant TB among younger people should be carefully monitored in urban areas, where Beijing strains and HIV coinfection are prevalent. Choosing an optimal treatment regimen on the basis of the results of drug susceptibility tests and monitoring of treatment adherence would minimize further development of drug resistance strains.

## Introduction

Resistance of *Mycobacterium tuberculosis* (MTB) to anti-tuberculosis (TB) drugs, particularly to isoniazid (INH) and rifampicin (RMP), which results in multidrug-resistant (MDR)-TB, presents a serious challenge in the control of TB worldwide [1,2]. The World Health Organization (WHO) estimates that the prevalence of MDR-TB varies from 0% to 65.1% across the world [1]. Despite progress in disease surveillance, more than 80% of MDR-TB patients are unaware of their disease status, indicating that the transmission status of MDR-TB is mostly unknown in high-TB burden countries [1].

Drug-resistant TB, including MDR-TB, develops as a result of inadequate treatment of an individual who was initially infected with a fully or partly sensitive strain or by direct transmission of a drug-resistant strain from one individual to another [3]. Although previous treatment is the strongest risk factor of MDR-TB, other risk factors such as younger age, male gender, and human immunodeficiency virus (HIV) coinfection have also been reported [4-6]. Further analysis may provide information on the dynamics of its transmission and better countermeasures against increasingly drug-resistant TB.

Viet Nam is one of the 22 countries with a high TB burden and is one of the 27 countries with a high MDR-TB burden [1]; the prevalence of any drug resistance and MDR-TB among newly diagnosed cases in a 2006 countrywide survey was 30.7% and 2.7%, respectively [7]. Although drug resistance, including MDR, and potential risk factors have been investigated in some areas [8-10], host-, pathogen-, and environment-related factors, such as patients’ HIV status; residential area; and genotypes of the MTB isolates, have not been comprehensively assessed in Viet Nam. We conducted this study to estimate the status of primary anti-TB drug resistance, including MDR, among newly diagnosed TB patients in Hanoi, the capital and second largest city of Viet Nam, and to investigate the role of the above risk factors in resistance to each of the first-line drugs.

## Materials and Methods

### Ethics statement

Written informed consent was obtained from each participant. In the case of minors, the parents provided written informed consent. This study was approved by the ethical committees of the Ministry of Health, Viet Nam, and National Center for Global Health and Medicine, Japan, respectively.

### Study sites, recruitment of patients, and sample collection

As part of our prospective study project, we included 7 of the 14 districts in Hanoi as the catchment area, where more than half of new smear-positive TB patients in the city were diagnosed and treated in the area during the study period. Among the districts, two were located in the old city area established before 1954 and had a population density that ranged from 25,000 to 26,000 individuals /km^2^ in 2009. As such, they were categorized as “old urban” areas. The remaining five districts were originally regarded as suburban areas. Of these, three were recently upgraded to urban areas on the basis of rapid economic development and had a population density that ranged from 2,800 to 5,300 individuals/km^2^, although the migrating population was not counted. We categorized these three areas as “new urban.” The two other areas remained “suburban,” and their population densities ranged from 1,500 to 2,500 individuals/km^2^.

Patients were considered eligible if they were 16 years or older, resided in the abovementioned catchment areas, suffered from smear-positive pulmonary TB without a history of TB treatment, and agreed to participate in this study. Eligible patients who visited the local TB care units were recruited consecutively from July 2007 to March 2009. Information about no previous TB treatment was based on interviews conducted by pre-trained health care staff and medical records kept for registration with the National TB Program in district TB centers.

Before initiating anti-TB treatment, sputum specimens were cultured and subjected to identification of MTB, drug susceptibility tests, and DNA extraction for molecular typing. Blood samples were obtained for HIV testing and complete blood count. Bacterial load estimated in sputum smear was used to assess the severity of the disease.

### Identification of MTB and drug susceptibility testing

After undergoing solid cultures on Löwenstein–Jensen media, MTB isolates from sputum specimens were subjected to a niacin test. For drug susceptibility testing, the WHO standard proportional method was used to identify resistance to INH, RMP, streptomycin (SM), and ethambutol (EMB) [11]. The test media contained INH (0.2 µg/mL), RMP (40 µg/mL), SM (4 µg/mL), and EMB (2 µg/mL). Resistance to pyrazinamide (PZA) was tested using a pyrazinamidase assay, in which pyrazinamidase activity was determined using Wayne’s method with minor modifications [12]. The H37Rv strain of MTB, which is susceptible to PZA and positive for pyrazinamidase, was used as the positive control. The BCG strain of *M. bovis*, which is resistant to PZA and negative for pyrazinamidase, served as the negative control.

### Molecular genotyping

Spoligotyping was performed to confirm the presence of Beijing strains and to identify sublineages of non-Beijing strains using a spoligotyping kit (Ocimum Biosolutions LLC, Houston, TX, USA), according to the standard protocol [13]. Classification of the spoligotype family was based on the international database, SpolDB4 [14].

We analyzed a single-nucleotide polymorphism at the 3284855 position using real-time polymerase chain reaction to further confirm the presence of Beijing strains [15].

Variable numbers of tandem repeats (VNTR) analysis was conducted for all strains using the international standard 15 mycobacterial interspersed repetitive unit (MIRU)-VNTR proposed by Supply et al. [16], with the exception of DNA samples with ambiguous results. The copy number of each locus of the H37Rv strain was used as to confirm the different definition in VNTR analysis. The copy numbers in MIRUs-4, 10, 16, 26, 31, and 40; ETRs-A and C; and VNTRs-2163b, 4052, 1955, 2401, 4156, 0424, and 3690 were defined as 3-3-2-3-3-1-3-4-5-5-2-2-2-2-5, respectively. We defined each cluster by complete match of the VNTR profile. To confirm the appropriateness of each cluster, spoligotyping patterns were also considered. The clustering rate was calculated as described elsewhere [17].

### Statistical analysis

The chi-squared test was used to compare the proportions between drug-sensitive and drug-resistant groups. The logistic regression models were used to evaluate potential risk factors for drug resistance, and adjusted odds ratios (aORs) and 95% confidence intervals (CIs) were calculated. Therein, each drug-resistance pattern was set as an outcome variable, and factors that could affect the pattern were chosen as independent variables. For RMP resistance and MDR, only variables with biological significance and with significant associations in univariate analysis were included in the multivariate models, because the number of outcome variables was limited. Statistical analysis was performed using Stata version 11 (StataCorp, College Station, TX, USA), and *P* < 0.05 was considered to be statistically significant.

## Results

### Study samples and patient characteristics

**Figure 1:**
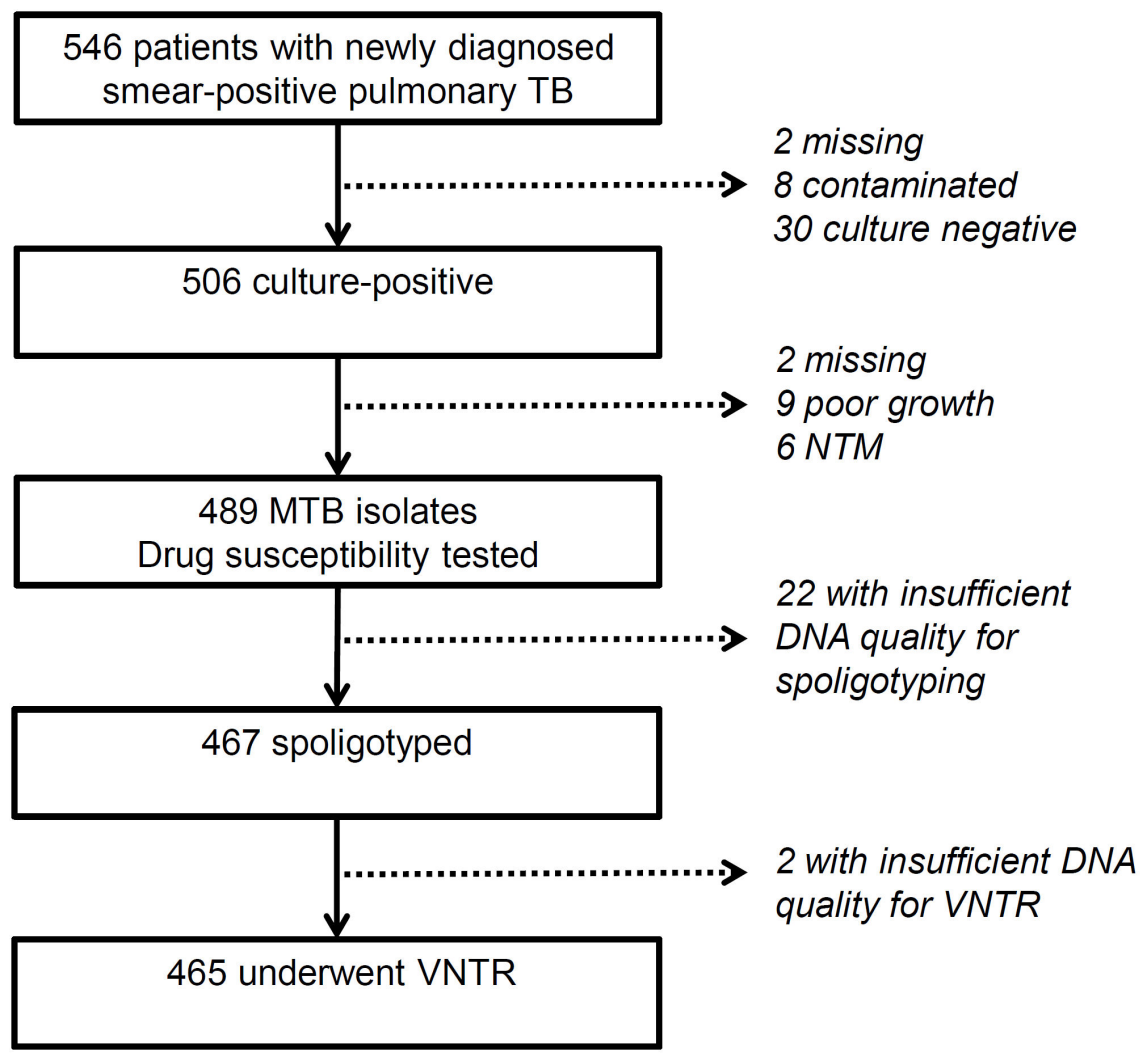
Study flow. TB: tuberculosis; MTB: *Mycobacterium tuberculosis*; NTM: nontuberculous mycobacterium; VNTR: variable numbers of tandem repeats; DNA: deoxyribonucleic acid.

In total, 546 newly diagnosed smear-positive pulmonary TB patients were recruited. From 506 culture-positive cases, microbial isolates were collected from 495 patients (97.8%), of which six were infected with nontuberculous mycobacteria. As a result, 489 MTB isolates were tested for drug susceptibility. Because of insufficient quality of the extracted DNA samples, 467 MTB isolates further underwent spoligotyping and 465 underwent VNTR typing (Figure 1). The median age was 38.6 years (range = 16.6–85.4), the proportion of male patients was 78.9%, and HIV coinfection was observed in 9.0% of the patients (Table 1).

**Table 1:** Characteristics of the study population (*n* = 489).

		**Number**	**%**
Age (median, range)		(38.6,	16.6–85.4)
Gender	Male	386	78.9
	Female	103	21.1
Body mass index	<16	70	14.3
	16–18.4	201	41.1
	18.5–24.9	213	43.6
	≥25	4	0.8
	Not available	1	0.2
Residential area	Suburban	100	20.4
	New urban	228	46.6
	Old urban	161	32.9
Smoking habit	Smoker	189	38.7
	Ex-smoker	134	27.4
	Nonsmoker	165	33.7
	No answer	1	0.2
HIV status	Positive	44	9.0
	Negative	443	90.6
	Not available	2	0.4

HIV: human immunodeficiency virus

### Prevalence and patterns of resistance to INH, SM, RMP, EMB, and PZA

Of the 489 MTB isolates, 60.9% were fully sensitive to INH, SM, RMP, and EMB. INH resistance was observed in 138 isolates (28.2%), which included 49 (10.0%) isolates of INH monoresistance; SM resistance was also observed in 138 isolates (28.2%), which included 50 isolates of SM monoresistance (10.2%), and the rest were mostly the combination of INH and SM resistance (Table 2). Primary resistance to RMP was detected in 24 isolates (4.9%), and 22 isolates were MDR-TB, which accounted for 4.5% of all isolates; most of these were also SM resistant. EMB resistance was not frequent (2.9%). The pyrazinamidase assay showed negative results for 12 isolates (2.5%), indicating resistance to PZA. The proportion of PZA resistance among MDR cases was significantly higher than that in non-MDR cases (13.6%, 95% CI 2.9–34.9 vs. 1.9%, 95% CI 0.9–3.6; *P* = 0.001; Table not provided).

**Table 2:** Patterns of INH, SM, RMP, and EMB resistance (*n* = 489).

**Pattern**		**Number**	**%**
Sensitive with all drugs		**298**	**60.9**
Any resistance	**Total**	**191**	**39.1**
	INH	138	28.2
	RMP	24	4.9
	SM	138	28.2
	EMB	14	2.9
Monoresistance	**Total**	**101**	**20.7**
	INH	49	10.0
	RMP	2	0.4
	SM	50	10.2
	EMB	0	0.0
Polyresistance, non-MDR	**Total**	**68**	**13.9**
	INH + SM	65	13.3
	INH + EMB	1	0.2
	INH + SM + EMB	1	0.2
	RMP + SM	0	0.0
	RMP + EMB	0	0.0
	RMP + SM + EMB	0	0.0
	SM + EMB	1	0.2
MDR	**Total**	**22**	**4.5**
	INH + RMP	1	0.2
	INH + RMP + EMB	0	0.0
	INH + RMP + SM	10	2.1
	INH + RMP + EMB + SM	11	2.2

INH: isoniazid; RMP: rifampicin; SM: streptomycin; EMB: ethambutol; MDR: multidrug resistance

### Distribution of MTB lineages and clusters of drug-resistant isolates

Among 467 MTB isolates spoligotyped, the Beijing genotype was most frequently observed [272 isolates (58.2%)]. The East African-Indian (EAI) lineage ranked as the second most frequently observed genotype [93 isolates (19.9%)], of which 84 isolates showed the EAI5 genotype and 9 showed a Vietnamese genotype (EAI4_VNM) (Table not provided). Among 21 of the 22 MDR-MTB strains available for spoligotyping, 15 (71.4%) were of Beijing genotype, 3 (14.3%) were of EAI genotype, and the remaining 3 (14.3%) showed unclassified non-Beijing genotypes but closely resembled EAI4 or 5, according to the spoligotyping database (Table 3).

**Table 3:** Characteristics of MDR-TB patients.

**No.**	**Gender, age**	**Residential area**	**HIV**	**DR pattern**	**MTB spoligotype**	**VNTR pattern**	**Clustered among MDR cases**	**Clustered among all cases**
138	M, 40	Old urban	Neg.	IRS	Beijing	2336**4**3446**8**44243	Yes (cluster I)	Yes (cluster I)
294	M, 22	Old urban	Neg.	IRSE	Beijing	2336**4**3446**8**44243	Yes (cluster I)	Yes (cluster I)
166	M, 50	Old urban	Neg.	IRS	Beijing	2336**5**3446**7**44243	Yes (cluster II)	Yes (cluster II)
347	F, 18	Suburban	Neg.	IRS	Beijing	2336**5**3446**7**44243	Yes (cluster II)	Yes (cluster II)
356	M, 30	New urban	Neg.	IRSE	Beijing	2336**5**3446**7**44243	Yes (cluster II)	Yes (cluster II)
239	M, 43	New urban	Neg.	IRSE	Unclassified	64224**5**742652124	Yes (cluster III)	Yes (cluster III)
256	M, 34	New urban	Pos.	IRSE	Unclassified	64224**5**742652124	Yes (cluster III)	Yes (cluster III)
48	F, 55	Old urban	Neg.	IRS	Beijing	233753447534443	Yes (cluster IV)	Yes (cluster IV)
449	M, 29	Old urban	Pos.	IRS	Beijing	233753447534443	Yes (cluster IV)	Yes (cluster IV)
205	M, 52	Suburban	Neg.	IRSE	Beijing	233751445854242	No	Yes (cluster V)
474	M, 26	New urban	Neg.	IR	Beijing	223753445854243	No	Yes (cluster VI)
36	M, 44	Old urban	Neg.	IRSE	Beijing	243753N42344335	No	No
69	M, 35	New urban	Neg.	IRS	Beijing	233753446754243	No	No
126	M, 26	New urban	Pos.	IRSE	Beijing	233751545854242	No	No
236	M, 34	Suburban	Pos.	IRS	EAI5	632253742692122	No	No
368	M, 40	New urban	Neg.	IRS	Beijing	232543443844443	No	No
409	M, 30	New urban	Pos.	IRSE	Beijing	233455444832423	No	No
489	M, 44	Old urban	Neg.	IRS	Beijing	223753445864243	No	No
528	M, 55	New urban	Neg.	IRSE	Unclassified	642245**4**42652124	No	No
16	M, 62	Old urban	Neg.	IRSE	N/A	N/A	N/A	N/A
264	M, 36	New urban	Pos.	IRSE	EAI5	N/A	N/A	N/A
333	M, 31	New urban	Pos.	IRS	EAI5	N/A	N/A	N/A

HIV: human immunodeficiency virus; MDR-TB: multidrug-resistant tuberculosis; DR: drug-resistant; VNTR: variable numbers of tandem repeats; M: male; F: female; IR: resistant to isoniazid and rifampicin; IRS: resistant to isoniazid, rifampicin, and streptomycin; IRSE: resistant to isoniazid, rifampicin, streptomycin, and ethambutol; Neg: negative; Pos: positive; MTB: *Mycobacterium tuberculosis*; N (in “VNTR pattern” column): polymerase chain reaction negative; EAI: East African–Indian; N/A: not available.

Of the 465 isolates, in which both spoligotype and VNTR patterns were available, 257 (55.3%) were clustered strains belonging to 55 clusters, indicating that the clustering rate was 43.4% [(257−55)/465]. The proportion of clustered strains was significantly higher in the group with any drug resistance than in the fully-sensitive group [112/178 (62.9%) vs. 145/287 (50.5%), *P* = 0.009]. Of the 22 MDR isolates, spoligotype and VNTR patterns of MTB were available in 19. Eleven (57.9%) of them belonged to six clusters, I–VI, as determined by a comparison of genotyping patterns observed in the 465 tested isolates, and clusters II and IV were the first (9.5%) and second (3.4%) largest clusters among them (Table not provided). MDR strains in the largest cluster II were observed in all of the old, new, and suburban areas. The VNTR patterns of the clusters I and II were different only in 2 of the 15 loci tested (Table 3).

### Factors associated with drug-resistant TB

The logistic regression models were used to identify factors associated with drug resistance. Factors that were analyzed included gender, age, body mass index (BMI), smoking behavior, the patient’s residential area, MTB load in the sputum smear before treatment, HIV status, the number of blood lymphocytes, MTB lineage, and clustered strains. Univariate and multivariate analyses (Tables 4 and 5) revealed that age less than 45 years, living in a new or old urban area, and being infected with Beijing strains were significantly associated with any drug resistance (aOR = 1.72, 95% CI 1.11–2.66; 2.06, 1.17–3.62; 2.14, 1.17–3.91; and 1.86, 1.21–2.87, respectively). However, living in an old urban area and being infected with Beijing strains or clustered strains were significantly associated with INH resistance (aOR = 2.23, 95% CI 1.15–4.35; 1.91, 1.18–3.10; and 1.69, 1.06–2.69, respectively), and being a smoker or infection with the Beijing MTB strain showed significant association with SM resistance (aOR = 2.47, 95% CI 1.18–5.16; 2.10, 1.29–3.40, respectively) (Table 5). Younger age was significantly associated with INH and SM resistance in univariate analysis (OR = 1.85, 95% CI 1.21–2.83; 1.77, 1.16–2.69, respectively) (Table 4), but these associations were not significant in multivariate analysis (aOR = 1.59, 95% CI 0.98–2.58; 1.56, 0.97–2.52, respectively) (Table not provided).

**Table 4:** Univariate analysis using the logistic regression model of the associations between potential risk factors and drug resistance (*n* = 489).

		**Any drug resistance**		**INH resistance**		**SM resistance**		**RMP resistance**		**MDR**	
		**OR**	**95% CI**	**OR**	**95% CI**	**OR**	**95% CI**	**OR**	**95% CI**	**OR**	**95% CI**
Age (in years)	≥45	1.00		1.00		1.00		1.00		1.00	
	<45	**1.85**	**1.26–2.71**	**1.85**	**1.21–2.83**	**1.77**	**1.16–2.69**	2.53	0.93–6.90	2.25	0.82–6.21
Sex	Male	1.00		1.00		1.00		1.00		1.00	
	Female	0.72	0.45–1.14	1.06	0.65–1.71	0.63	0.38–1.06	0.33	0.08–1.42	0.36	0.08–1.58
Smoking*	No	1.00		1.00		1.00		1.00		1.00	
	Yes	**1.69**	**1.14–2.51**	1.28	0.84–1.96	**2.00**	**1.28–3.14**	1.89	0.69–5.18	1.67	0.60–4.64
HIV status	Negative	1.00		1.00		1.00		1.00		1.00	
	Positive	**1.98**	**1.06–3.70**	**2.07**	**1.10–3.89**	**2.30**	**1.22–4.31**	**4.74**	**1.85–12.16**	**5.40**	**2.07–14.07**
Number of lymphocytes (cells/mm^3^)											
	≥1,000	1.00		1.00		1.00		1.00		1.00	
	<1,000	**1.74**	**1.01–3.01**	1.61	0.91–2.85	1.63	0.92–2.89	1.99	0.71–5.54	2.23	0.79–6.30
Smear**		0.91	0.76–1.11	0.90	0.73–1.11	0.88	0.72–1.09	0.89	0.57–1.37	0.92	0.58–1.44
MTB strain	Non-Beijing	1.00		1.00		1.00		1.00		1.00	
	Beijing	**2.00**	**1.35–2.95**	**2.11**	**1.37–3.26**	**1.95**	**1.26–3.00**	1.68	0.68–4.16	1.84	0.70–4.83
Clustered	No	1.00		1.00		1.00		1.00		1.00	
	Yes	**1.66**	**1.13–2.44**	**2.08**	**1.36–3.20**	1.16	0.77–1.75	1.08	0.45–2.62	1.12	0.44–2.83
BMI	18.5–24.9	1.00		1.00		1.00		1.00		1.00	
	<16	0.85	0.49–1.49	0.72	0.38–1.36	0.95	0.52–1.74	0.69	0.19–2.49	0.82	0.22–3.04
	16–18.4	1.02	0.68–1.51	0.99	0.65–1.51	1.03	0.67–1.58	0.64	0.26–1.57	0.76	0.30–1.93
	≥25	1.54	0.21–11.11	2.44	0.34–17.67	0.85	0.09–8.33	-		-	
Residential area											
	Suburban	1.00		1.00		1.00		1.00		1.00	
	New urban	**1.97**	**1.18–3.29**	1.70	0.96–3.03	1.42	0.82–2.45	1.64	0.45–6.01	1.64	0.45–6.01
	Old urban	**1.98**	**1.15–3.40**	**2.15**	**1.18–3.91**	1.38	0.78–2.46	2.14	0.57–7.98	1.69	0.44–6.53

INH: isoniazid; SM: streptomycin; RMP: rifampicin; MDR: multidrug-resistance; HIV: human immunodeficiency virus; BMI: body mass index; MTB: *Mycobacterium tuberculosis*; OR: odd ratios; 95% CI: 95% confidence interval

* Includes ex-smoking.

** OR per unit change of smear positivity (scanty, 1+, 2+, 3+).

Bold type indicates significant associations.

**Table 5:** Results of multivariate analysis using the logistic regression model on the associations between potential risk factors and drug resistance (*n* = 489).

**Factors**			**Number (%)**	**Multivariate**	
				**aOR**	**95% CI**
Any drug resistance*					
	Age (in years)	≥45	58/191 (30.4)	1.00	-
		<45	133/298 (44.6)	**1.72**	**1.11–2.66**
	Smoking**	No	51/165 (30.9)	1.00	-
		Yes	139/323 (43.0)	**1.87**	**0.99–3.49**
	Residential area	Suburban	27/100 (27.0)	1.00	-
		New urban	96/228 (42.1)	**2.06**	**1.17–3.62**
		Old urban	68/161 (42.2)	**2.14**	**1.17–3.91**
	MTB strain	Non-Beijing	57/195 (29.2)	1.00	-
		Beijing	123/272 (45.2)	**1.86**	**1.21–2.87**
INH resistance*					
	Residential area	Suburban	19/100 (19.0)	1.00	-
		New urban	65/228 (28.5)	1.60	0.85–3.02
		Old urban	54/161 (33.5)	**2.23**	**1.15–4.35**
	MTB strain	Non-Beijing	38/195 (19.5)	1.00	-
		Beijing	92/272 (33.8)	**1.91**	**1.18–3.10**
	Clustered	No	41/207 (19.8)	1.00	-
		Yes	87/258 (33.7)	**1.69**	**1.06–2.69**
SM resistance*					
	Smoking**	No	32/165 (19.4)	1.00	-
		Yes	105/323 (32.5)	**2.47**	**1.18–5.16**
	MTB strain	Non-Beijing	39/195 (20.0)	1.00	-
		Beijing	89/272 (32.7)	**2.10**	**1.29–3.40**
RMP resistance***					
	HIV	Negative	17/443 (3.8)	1.00	-
		Positive	7/44 (15.9)	**5.42**	**2.07–14.14**
	MTB strain	Non-Beijing	7/195 (3.6)	1.00	-
		Beijing	16/272 (5.9)	1.67	0.67–4.20
MDR***					
	HIV	Negative	15/443 (3.4)	1.00	-
		Positive	7/44 (15.9)	**6.23**	**2.34–16.58**
	MTB strain	Non-Beijing	6/195 (3.1)	1.00	-
		Beijing	15/272 (5.5)	1.84	0.69–4.90

INH: isoniazid; SM: streptomycin; RMP: rifampicin; MDR: multidrug-resistance; TB: tuberculosis; HIV: Human immunodeficiency virus; aOR: adjusted odd ratios; 95% CI: 95% confidence interval

* Only factors showing significant associations were shown.

** Included ex-smoking.

*** The final model included biologically significant variables (MTB lineage) and variables showing significant associations (HIV status) in univariate analysis.

Bold type indicates significant associations.

Multivariate analyses revealed that only HIV coinfection was significantly associated with RMP resistance (aOR = 5.42, 95% CI 2.07–14.14) and MDR (aOR = 6.23, 95% CI 2.34–16.58) (Tables 4 and 5).

## Discussion

We found that the proportion of drug-resistant cases, including MDR, was considerably high among newly diagnosed smear-positive culture-positive pulmonary TB patients residing in Hanoi city. Depending on the type of drug resistance, the drug resistance-associated risk factors showed a pronounced variation and revealed complicated aspects in a large city. The majority of MDR-TB cases revealed that infection with Beijing strains was predominantly spread in this area, while non-Beijing MDR strains were also observed.

INH or SM resistance was not uncommon, and most RMP-resistant strains were also associated with SM and INH resistance, resulting in MDR. These findings were consistent with a previous report in Ho Chi Minh city in Viet Nam [9]. The high prevalence of primary resistance to INH and SM (28.2% and 28.2%, respectively) and moderate prevalence of RMP resistance and MDR (4.9% and 4.5%, respectively) shown in our study might be considered noteworthy, when comparing with those of South East Asian region (10.3%, 8.9%, 3.4%, and 2.8%) [7], and of China (16.0%, 27.7%, 6.7%, and 5.7%) [18]. In this situation, the use of a regimen with RMP for only 2 months of the intensive phase, which is still accepted in Viet Nam, may pose the risk for poor treatment outcome [19] and accumulation of further drug resistance [20].

The association between younger age and anti-TB drug resistance has been reported previously [9,21]. The results of univariate and multivariate analyses performed in our study indicate that primary drug resistance among the younger population may be confounded by the recent transmission of Beijing strains [9,22]. In the current study, living in an old urban area and infection with clustered strains were associated with INH, but not SM, resistance, suggesting that the transmission of INH-resistant strains is concentrated in areas with a high population density, whereas SM-resistant strains are spreading more diffusely throughout the city. Initially, SM was used for treatment of wound infections during the war in Viet Nam in the early 1950s, which may partly explain the widespread development of SM-resistant nonclustered strains, whereas INH was first circulated in 1960s, and RMP was introduced at around 1975 [23,24]. The Beijing genotype was significantly associated with resistance to any drug, INH, and SM, but it was not associated with either RMP resistance or MDR. A direct role of Beijing strains in drug resistance remains controversial [22,25-27].

The spoligotype and VNTR analyses demonstrated that any-drug resistant strains showed a higher tendency for clustering than fully-sensitive strains; and almost half of the MDR strains were clustered and presumably derived from common infection sources or infection with different sources sharing ancestors [16,28]. Three of the MDR strains (13.6%) belonged to the largest Beijing cluster, accounting for approximately 10% of the study population. Although the Beijing genotype was predominant among clustered MDR strains, three non-Beijing genotype strains were closely related to each other based on their VNTR patterns and showed unclassified spoligo patterns resembling EAI5 or EAI4_VNM, a possibly indigenous MTB subtype mainly observed in Viet Nam. Research into the origin and transmission dynamics of these variant MDR strains, as well as their molecular characteristics, may be important, because it is generally believed that the EAI lineage has conferred significantly less drug resistance compared with other genotypes in Asian countries [29,30].

HIV coinfection was significantly associated with only RMP resistance and MDR in multivariate analysis, although it showed significant associations with all types of drug resistance in univariate analysis. This independent association with RMP resistance and MDR has also been reported in other studies [31,32], including one in the northern area [10], but was not observed in a study of the southern area of Viet Nam [9]. The southern study was conducted between 1998 and 2000, when HIV prevalence was low in Viet Nam [33]. This may explain the lower percentage of HIV, compared with ours (2.8% vs. 9.0%), resulting in a low statistical power (20%) [9]. In Hanoi, approximately 25% of injecting drug users tested were HIV positive [31]. Drug use is a risk factor for nonadherent treatment, and it promotes development of drug resistance [34], thus increasing the chance of resistance transmission among the group. HIV coinfection is also associated with pharmacokinetic alteration of RMP, resulting in a 39% reduction of drug concentration [35]. The decreased bioavailability of RMP may contribute to the development of RMP resistance as well. In addition, HIV-coinfected TB patients receiving antiretroviral treatment often suffer from the adverse effects of RMP when an alternative drug is not available, which may cause poor treatment outcomes [36] and facilitate drug resistance. The negative effect of HIV coinfection on RMP resistance, together with the recent spread of Beijing strains associated with INH resistance, may pose a combined risk for the acquisition and transmission of MDR-TB in a large city like Hanoi.

SM resistance was independently associated with smoking, after adjusted for HIV coinfection. The reason for this association is unknown, although smoking is known to be associated with TB [37]. The proportion of PZA resistance tested using the pyrazinamidase assay was low among the total study population [38]. Nevertheless, the proportion of PZA resistance was significantly higher in the MDR group than that in the non-MDR group, indicating a need for evaluation of the susceptibility of MTB strains to this drug.

The clustering rate in Hanoi (43.4%) was high, presumably because our study was conducted in a capital city with high population density and enrolled only patients with smear-positive pulmonary TB. Others have reported relatively lower clustering rates (28.3% in China [39], 37.7% in Zambia [40], and 16.8% in Uganda [41]). However, these studies were conducted in peripheral areas (Zambia) or enrolled patients with smear-negative pulmonary TB (China, Uganda). In addition, it is known that the resolution of 15 MIRU-VNTR for Beijing strains is suboptimal and may overestimate the clustering rate. Addition of more loci to the standard VNTR loci may increase the resolution in a setting where Beijing-genotype strains prevail [42]. Nevertheless, the data can be analyzed using the standard 15 MIRU-VNTR typing method first, since it has been used internationally for a long time [39-41,43,44].

Our study has some limitations. First, we did not have enough information about direct epidemiologic links among clustered patients. In high TB burden countries, however, a TB outbreak is difficult to identify. In addition, we may not have analyzed all representative isolates in Hanoi city. However, the seven districts participating in this study cover old urban, new urban, and suburban areas in this city, and analysis of a relatively large number of isolates definitely provided information that would be useful in the management of drug-resistant TB. Despite the aforementioned limitations, we investigated a variety of host-, bacteria-, and environment-related factors and developed a multidimensional picture of the status of drug-resistance in the studied area.

In conclusion, the transmission status of drug-resistant TB in a large city with a high proportion of Beijing strains, particularly in HIV-prevalent areas, should be carefully monitored to avoid an increase in the incidence of MDR and generation of extensively drug-resistant TB. Drug susceptibility testing should be considered. On the basis of the results, an optimal treatment regimen, together with intensive monitoring of treatment adherence, is suggested to avoid further increases in drug resistance.
